# Spatio-temporal pattern analysis for evaluation of the spread of human infections with avian influenza A(H7N9) virus in China, 2013–2014

**DOI:** 10.1186/s12879-017-2781-2

**Published:** 2017-10-24

**Authors:** Wen Dong, Kun Yang, Quanli Xu, Lin Liu, Juan Chen

**Affiliations:** 1 School of Information Science and Technology, Yunnan Normal University, Kunming, Yunnan China; 2 School of Tourism and Geographic Science, Yunnan Normal University, Kunming, Yunnan China; 3 GIS Technology Engineering Research Centre for West-China Resources and Environment of Educational Ministry, Yunnan Normal University, Kunming, Yunnan China

**Keywords:** A(H7N9) human infections, Spreading pattern, Directional trend, Spatio-temporal clusters

## Abstract

**Background:**

A large number (*n* = 460) of A(H7N9) human infections have been reported in China from March 2013 through December 2014, and H7N9 outbreaks in humans became an emerging issue for China health, which have caused numerous disease outbreaks in domestic poultry and wild bird populations, and threatened human health severely. The aims of this study were to investigate the directional trend of the epidemic and to identify the significant presence of spatial-temporal clustering of influenza A(H7N9) human cases between March 2013 and December 2014.

**Methods:**

Three distinct epidemic phases of A(H7N9) human infections were identified in this study. In each phase, standard deviational ellipse analysis was conducted to examine the directional trend of disease spreading, and retrospective space-time permutation scan statistic was then used to identify the spatio-temporal cluster patterns of H7N9 outbreaks in humans.

**Results:**

The ever-changing location and the increasing size of the three identified standard deviational ellipses showed that the epidemic moved from east to southeast coast, and hence to some central regions, with a future epidemiological trend of continue dispersing to more central regions of China, and a few new human cases might also appear in parts of the western China. Furthermore, A(H7N9) human infections were clustering in space and time in the first two phases with five significant spatio-temporal clusters (*p* < 0.05), but there was no significant cluster identified in phase III.

**Conclusions:**

There was a new epidemiologic pattern that the decrease in significant spatio-temporal cluster of A(H7N9) human infections was accompanied with an obvious spatial expansion of the outbreaks during the study period, and identification of the spatio-temporal patterns of the epidemic can provide valuable insights for better understanding the spreading dynamics of the disease in China.

## Background

Since the first avian influenza A (H7N9) outbreak in human occurred in March 2013 in Zhejiang, H7N9 outbreaks became an emerging issue for China health: they have caused numerous disease outbreaks in domestic poultry and wild bird populations, and threatened human health severely [1–3]. Between March 2013 and December 2014, a total of 460 A(H7N9) human infections with severe respiratory illness and mortality have been reported to the World Health Organization [4].

Phylogenetic analysis of H7N9 demonstrated that the novel avian-origin influenza A(H7N9) virus was a novel triple reassortant of avian influenza A H7N3, A H7N9, and A H9N2 viruses [5–7]. The virus was previously found only in wild bird populations, but in this outbreak it has been proved to be a novel strain, the first of its kind ever found to infect humans [8]. Gene sequence studies have showed that the virus combines with human receptors more easily than with birds [9]. The virus can infect birds without symptoms, but it can cause severe respiratory illness and result in high mortality in human populations. In spite of the long presence of A(H7N9) human infections in China, the long-term spatiotemporal dynamics and geographic spread mechanisms of H7N9 outbreaks in China have not been well characterised. So it is necessary to investigate the transmission pattern of A(H7N9) human infections and identify the significant clustering of influenza A(H7N9) human cases. Understanding how A(H7N9) human infections spread in China can help controlling the epidemic in the future.

Standard deviational ellipse analysis has been used in some studies to investigate the directional trend and model the spread of outbreak over time [10–14], but the research on the directional trend of H7N9 is very limited, and so far there was only one former study used this method to explore the directional distribution of influenza A(H7N9) human cases in the Yangtze River Delta between 19 February 2013 and 18 April 2013 [15]. In addition, as an helpful adjunct for assessing the spatiotemporal distribution of an epidemic, the SatScan method has been undertaken in some previous studies to explore the spatio-temporal clustering of H7N9 outbreaks in China [3, 16]. Although some spatio-temporal clusters of H7N9 have been identified in these researches, the study periods are not long enough neither (the longest study in these papers was conducted between 19 February 2013 and 31 March 2014 [3]). However, the spreading of influenza A(H7N9) human infections is a dynamic development process and the spread mechanisms of the epidemic are still unclear till now, consequently these researches may not be adequate for seasonal H7N9 outbreaks in different epidemic phases, and further studies with more case data covering all seasons of a full year are still necessary to investigate the periodical spatiotemporal spreading pattern of H7N9.

In order to examine the ever-changing spreading pattern of H7N9 outbreaks, by using information on all influenza A(H7N9) human cases reported in China between March 2013 and December 2014, GIS-based standard deviational ellipse analyses and SaTScan-based retrospective space-time permutation scan statistics were conducted in different epidemic phases of China. The main purposes of this study were: (1) to investigate the epidemic characteristics and the directional trend of the H7N9 outbreaks, which can provide clues for understanding the spreading dynamics of the epidemic that have occurred in China in different phases. And (2) to identify the significant presence of spatial-temporal clustering of influenza A(H7N9) human cases during each phase, and this is helpful in confirming geographical locations and times of the year in which H7N9 outbreaks are most likely to occur, and then the government can apply control strategies specifically. As a complement to epidemiological studies, results of this study can provide valuable insights for better understanding the spreading dynamics of the disease, and hence improve the efficiency of future strategic planning in the prevention and control of H7N9 outbreaks.

## Methods

### Data management

In order to fully examine the spread of the H7N9 outbreaks in humans, the disease data consisted of 460 influenza A(H7N9) human cases reported in China from March 2013 through December 2014, which were aggregated at the municipal level, and the spatio-temporal attributes of each H7N9 human case were used, including the location (latitude, longitude) and the date of influenza A(H7N9) human cases.

We obtained information about the confirmed influenza A(H7N9) human cases from the World Health Organization website [4]. A Chinese topographic map (1:4,000,000 scales) available from the National Fundamental Geographic Information System of China [17] was used to visualize the reported influenza A(H7N9) human cases, the identified standard deviational ellipses and the spatio-temporal clusters of H7N9 outbreaks by using ArcGIS 10.1 software (ESRI Inc., Redlands, CA, USA).

### Epidemic phases

Based on the daily and monthly data of influenza A(H7N9) human cases reported in China from March 2013 to December 2014, the corresponding epidemic curves of H7N9 were created by R 3.1.3 software [18] to display the magnitude and temporal trend of H7N9 outbreak. According to the epidemic curves, we regarded a full time period as an epidemic phase, in which the number of influenza A(H7N9) human cases peaked and then decreased until the cases finally disappeared. Accordingly, a quantitative description of H7N9 transmission could be presented by displaying the staged dynamics of the standard deviational ellipse and the spatio-temporal clusters of the epidemic.

### Directional trend analysis

The standard deviational ellipse analysis can well measure the spatial distribution of geographic features around their geometric center, and provide useful information about feature dispersion and orientation [19]. How standard deviational ellipse works has been described in detail in ArcGIS Help 10.1 [20]. In this study, standard deviational ellipse analysis was carried out with one standard deviation in ArcGIS 10.1 (ESRI Inc., Redlands, CA, USA) to investigate the size and the movement of the H7N9 epidemic, and then to measure whether the distribution of influenza A(H7N9) human cases displays a directional trend during the three epidemic phases.

This analysis can create ellipse to summarize the spatial characteristics of the influenza A(H7N9) human cases: central tendency, dispersion, and directional trends. The ellipse shows the spatial spread of a set of point locations, and helps us to determine whether the distribution of these points is elongated and hence they spread with a particular orientation.

### Spatio-temporal cluster analysis

For identifying the spatio-temporal clusters of A(H7N9) human infections in each epidemic phase, retrospective space-time permutation scan statistics were applied by using SaTScan 9.4.2 [21]. The space-time permutation model only requires case data with exact spatial location and time, but no need about the background population at risk [22–24].

The scanning window in the space-time permutation model was a cylinder with the base representing space and the height representing time, respectively [25]. In this research the window moves both in time and in space to cover each possible time interval for each possible geographic location, and the model parameters for maximum spatial and temporal window sizes were set such that a cluster could include a maximum of 50% of all influenza A(H7N9) human cases. During the analysis, spatio-temporal clusters occur when an excess number of H7N9 outbreaks are observed, and the log-likelihood ratio statistic [26–28] was used to evaluate whether the cylinder contained a cluster or not. The scanning window with the maximum log-likelihood ratio was defined be the most likely cluster, and the other windows with statistically significant log-likelihood ratio were considered as secondary clusters, which were ranked by the value of their log-likelihood ratio [23]. The *P*-value for the detected clusters was evaluated by using Monte Carlo simulation [29]. Ensuring adequate precision, the number of Monte Carlo simulations was set at 999, and the time units of 5 days were used to reflect potential disease spread. For all analyses, the most likely cluster and non-overlapping secondary clusters with statistical significance of *P* < 0.05 were reported.

## Results

### Spacial distribution analysis

**Fig. 1:**
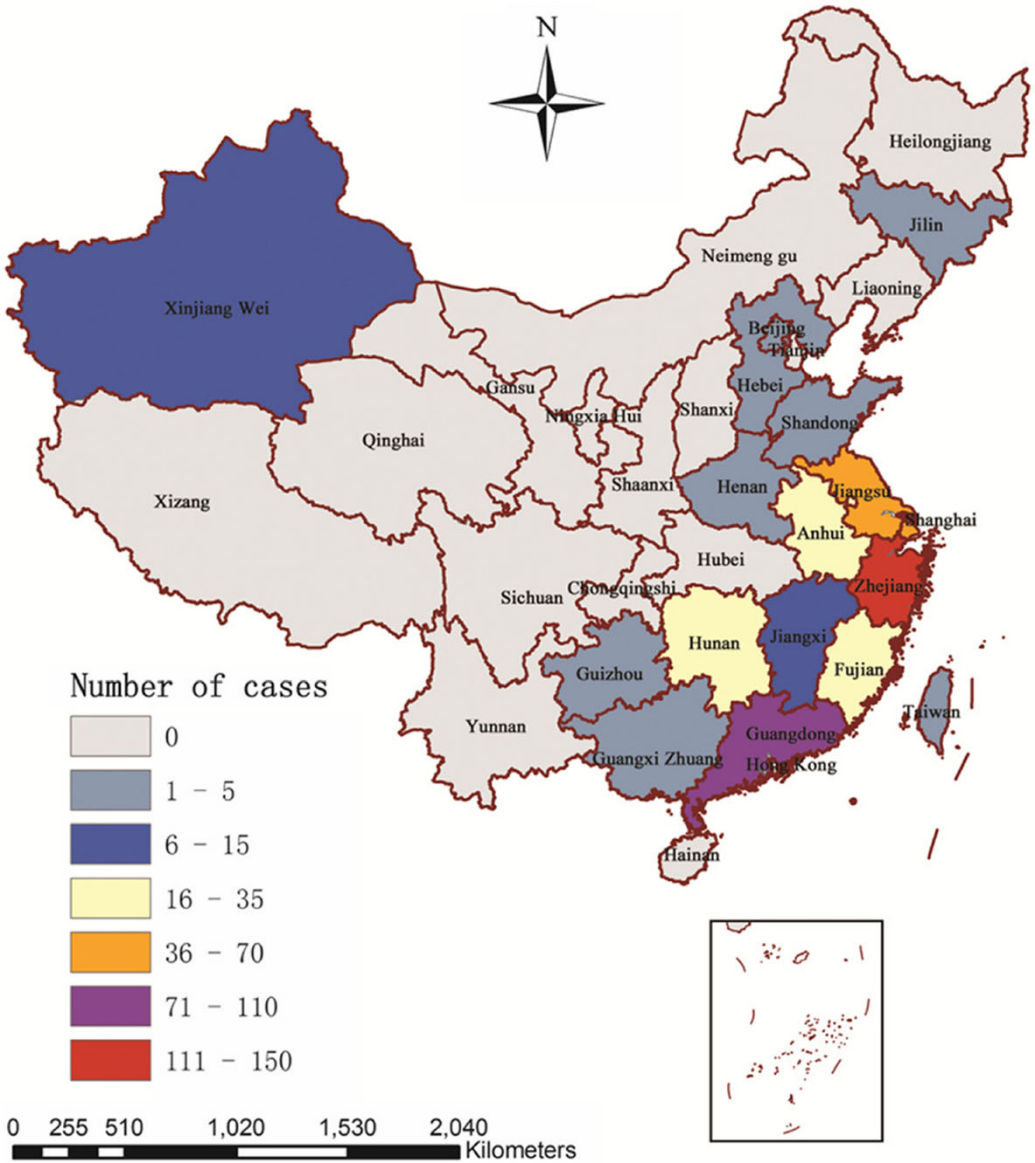
The spacial distribution of reported influenza A(H7N9) human cases in China between March 2013 and December 2014 (*n* = 460)

During the H7N9 epidemic period (March 2013 to December 2014, 658 days) studied in this paper, a total of 460 influenza A(H7N9) human cases occurred in 18 provinces, municipalities and autonomous regions of China. The spatial distribution of influenza A(H7N9) human cases is shown in Fig. 1. It clearly indicates that the distribution of A(H7N9) human infections was heterogeneous at the provincial level. The results show that the largest number of influenza A(H7N9) human cases was reported from Zhejiang (142), followed by Guangdong (104), Jiangsu (62) and Shanghai (42), all of them were located in the southeast coast of China, which accounted for about 76.1% of influenza A(H7N9) human cases (350/460).

### Epidemic phases

**Fig. 2:**
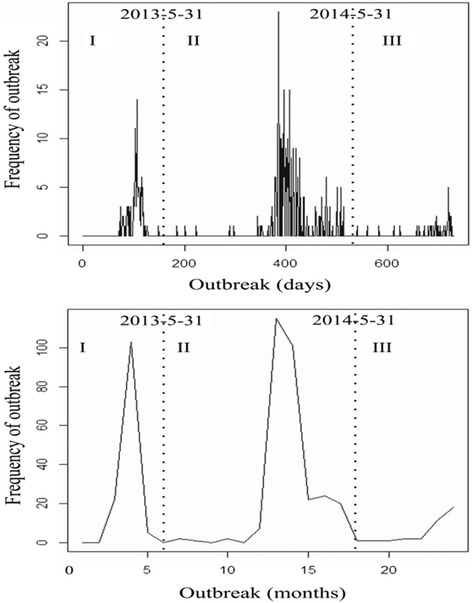
Epidemic curves for daily and monthly reported A(H7N9) human infections in China from March 2013 to December 2014. The three phases of the epidemic are indicated by dashed lines

The epidemic curve shows the temporal pattern of H7N9 outbreaks in humans from March 2013 to December 2014 in China, which consisted of three distinct phases (Fig. 2): phase I was from March 13, 2013 to May 31, 2013 (*n* = 130), with peak observed in April 16, 2013; phase II was from June 1, 2013 to May 31, 2014 (*n* = 294), with peak observed in January 20, 2014; phase III was from June 1, 2014 to December 31, 2014 (*n* = 36), with peak observed in December 21, 2014. From Fig. 2 it also can be seen that there was a similar seasonal pattern showed by different time scales of day and month: influenza A(H7N9) season generally began in early winter (November to December), and then the epidemic reached its peak between late winter and late spring (January to April), but declined in incidence between May and June and then entered a sustained low incidence period (July to October).

### Directional trend analysis

**Fig. 3:**
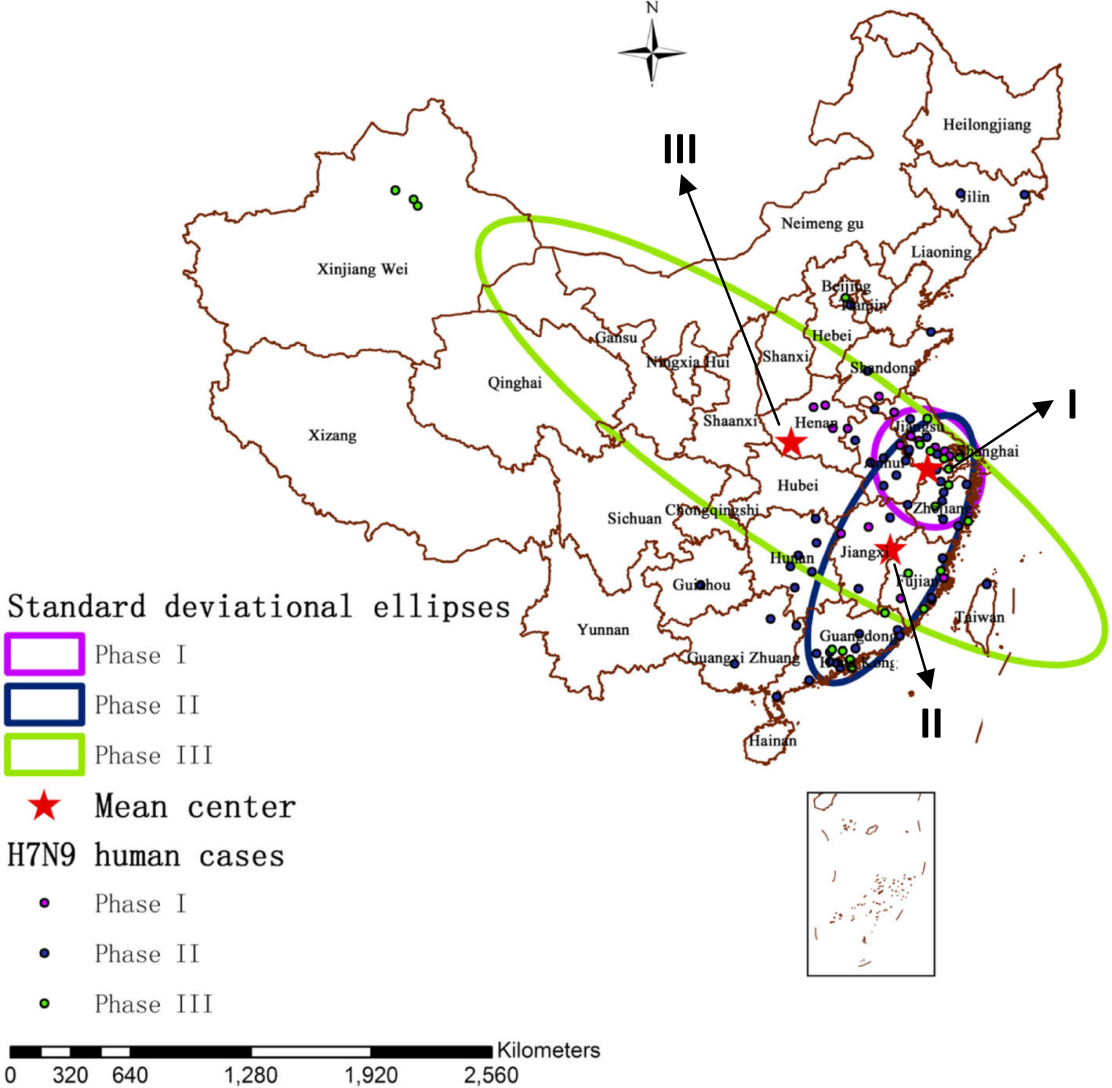
The mean centers and standard deviational ellipses of A(H7N9) human infections in three distinct phases in China during March 2013 and December 2014 (each colored ellipse represents a 1 standard deviation distribution of H7N9 outbreaks). Distribution of influenza A(H7N9) human cases by phases is also shown

Table 1 summarizes the characteristics of standard deviational ellipses: X coordinate (CenterX) and Y coordinate (CenterY) for the mean center; the standard distance of X-axis (XStdDist) and of Y-axis (YStdDist), in which the larger one is long axis and the smaller one is short axis; the orientation of the ellipse (Rotation). Fig. 3 illustrates clearly the mean centers and the directional trends of influenza A(H7N9) human cases in the three phases by standard deviational ellipses (1 standard deviation).

**Table 1:** Characteristics of standard deviational ellipses by phase

Phase	Location of the mean center		Ellipse size of the epidemics		Orientation of the ellipse
	CenterX (m)	CenterY (m)	XStdDist (km)	YStdDist (km)	Rotation (°from North)
1	1,383,592.750160	3,949,191.040550	325.181481980	277.821569251	152.621015
2	1,184,775.846750	3,516,687.088910	294.358161767	785.662350587	26.947956
3	656,953.359159	4,089,979.224960	1976.727605830	539.929271709	123.918912

In phase I, the mean center of the ellipse (x = 1,383,592.750160 m, y = 3,949,191.040550 m) was located in Xuancheng, Anhui province, and the ellipse was confined in eastern China, mainly including Jiangsu, Anhui, Zhejiang and Shanghai. Moreover, the ellipse showed a northwest-southeast elongation (long axis = 325.181481980 km, short axis = 277.821569251 km), with an angular rotation of 152.621015°.

In phase II, the spatial distribution of A(H7N9) human infections spread to southeast coastal areas, and the mean center of the ellipse (x = 1,184,775.846750 m, y = 3,516,687.088910 m) moved southwestward and finally located in Fuzhou, Jiangxi province, which was about 476.01 km southwest of the mean center of phase I. Consequently, the coverage of the ellipse in phase II also spread from the east to southeast coastal areas of China, mainly including Jiangsu, Anhui, Zhejiang, Shanghai, Jiangxi, Fujian, Guangdong, Hunan, and so on. In addition, the ellipse presented a southwest-northeast elongation (long axis = 785.662350587 km, short axis = 294.358161767 km), and the angular rotation was 26.947956°.

The H7N9 virus then spread rapidly from southeast coastal areas to central China, even dispersed to a slice of areas of western China during the third phase. In phase III, the mean center of the ellipse (x = 656,953.359159 m, y = 4,089,979.224960 m) moved northwestward and finally located in Nanyang, Henan province, which was about 779.27 km northwest of the mean center of phase II. Accordingly, the extent of the ellipse in phase III reached the maximum of the three phases, mainly including Taiwan, Fujian, Jiangxi, Zhejiang, Jiangsu, Anhui, Hubei, Henan, Shaanxi, Shanxi, Ningxia, Gansu, Hunan, and so on. Furthermore, the ellipse showed a northwest-southeast elongation (long axis = 1976.727605830 km, short axis = 539.929271709 km), with an angular rotation of 123.918912°.

### Spatio-temporal cluster analysis

Using the surveillance data of influenza A(H7N9) human cases collected in the three epidemic phases, spatio-temporal cluster analysis identified two most-likely clusters and three secondary clusters during the study period.

#### Spatio-temporal cluster in phase I

**Fig. 4:**
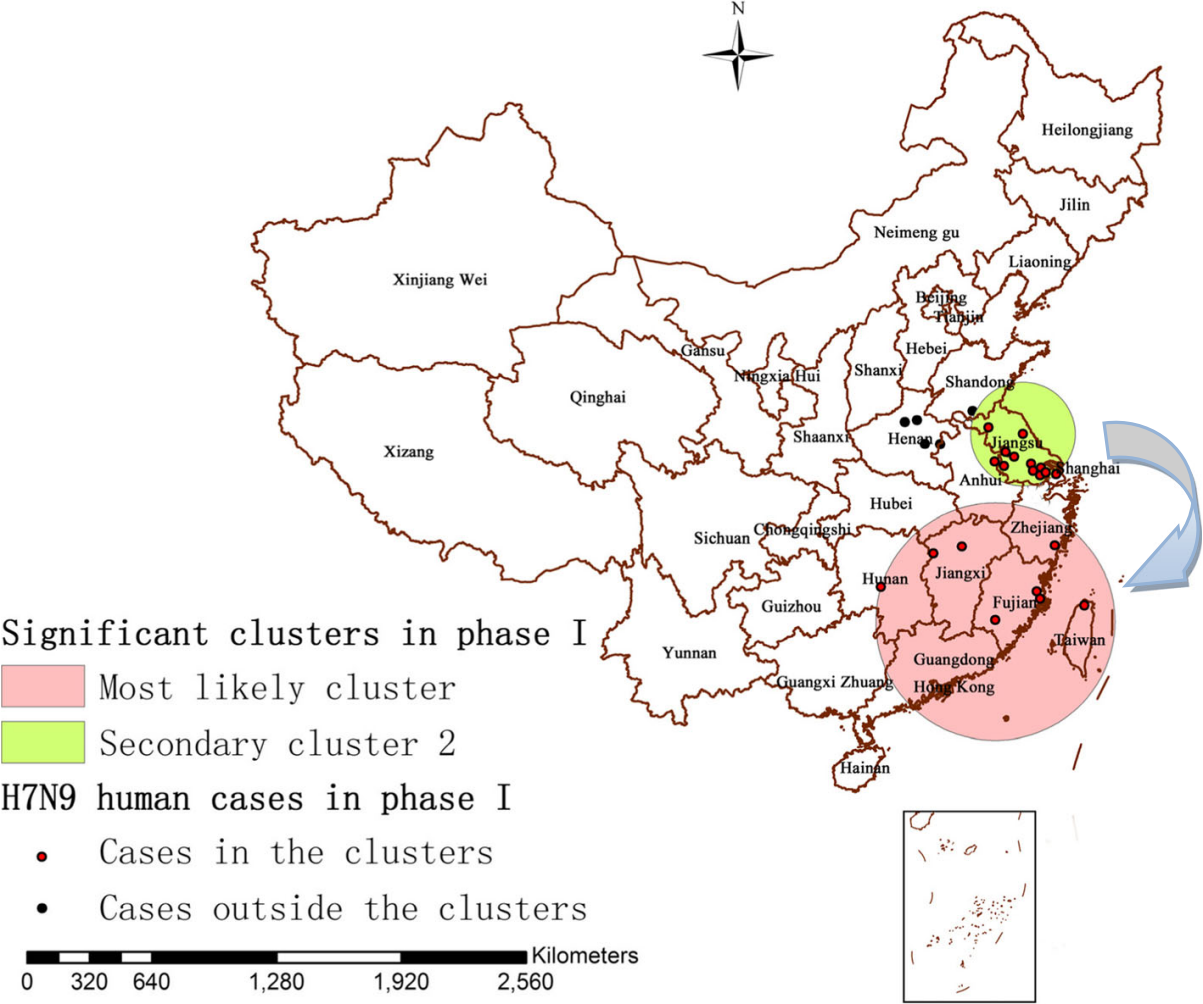
Significant spatio-temporal clusters (*P* < 0.05) of A(H7N9) human infections in China as identified by the space-time permutation scan statistic (phase I), which are illustrated by the most likely cluster (pink circle) and by a secondary cluster 2 (green circle). The blue arrow describes the chronological progression of clusters, and outbreaks represented by red and black dots are also shown

There were totally 31 localities and 130 influenza A(H7N9) human cases contained in the first epidemic phase. The identified statistically significant spatio-temporal clusters of A(H7N9) human infections in this period was displayed in Fig. 4, which overlaid with the correlated influenza A(H7N9) human cases in phase I. The scan test identified two significant spatio-temporal clusters of H7N9 outbreaks (*P* < 0.05) between March 13, 2013 and May 31, 2013 (79 days), a most likely cluster and a secondary cluster (Fig. 4 and Table 2), subsequently the null hypothesis could be rejected. The most likely cluster (*P* = 0.0000055) including eight localities was observed in the period from April 27, 2013 to May 11, 2013 with a radius of 596.94 km, which was detected in Fujian, Jiangxi, Taiwan, Zhejiang and Hunan provinces, southeast of China. Based on the time period and the area of the cluster, this cluster showed 10 influenza A(H7N9) human cases, which accounted for 7.70% of all the human cases of phase I (10/130), when only 1.38 human cases could be expected theoretically. Other, the secondary cluster 2 (*P* = 0.044) including 12 localities was located in Jiangsu, Anhui provinces and Shanghai municipality, Yangtze river delta region of eastern China, with a radius of 271.46 km. This cluster showed 31 influenza A(H7N9) human cases between March 13, 2013 and April 11, 2013, accounting for 23.85% of the total human cases occurred in this phase (31/130), whereas only 17.36 could be expected.

**Table 2:** Statistically significant spatio-temporal clusters of A(H7N9) human infections in China identified by use of the space–time permutation scan test with 5-days scanning unit (phase I)

Cluster	Most likely cluster	Secondary cluster 2
Location	Longyan, Fuqing, Fuzhou, Nanchang, Taiwan, Tonggu, Wenzhou, Shaoyang	Tianchang, Yancheng, Yangzhou, Jiangyin, Suqian, Nanjing, Wuxi, Changshu, Chuzhou, Suzhou, Kunshan, Shanghai
Province or region	Fujian, Jiangxi, Taiwan, Zhejiang, Hunan	Jiangsu, Anhui, Shanghai
Coordinates	25.12 N, 117.01 E	33.38 N, 120.13 E
Radius (km)	596.94 km	271.46 km
Time frame	2013 4/27–5/11	2013 3/13–4/11
Duration (days)	14	29
O^a^	10	31
E^b^	1.38	17.36
O / E^c^	7.22	1.79
LLR^d^ / Test statistic	11.451464	5.194351
P-value^e^	0.0000055	0.044

^a^Number of cases observed per cluster

^b^Number of cases expected per cluster

^c^Observed / Expected, Observed-to-expected ratio

^d^Log Likelihood ratio

^e^Statistical significance was evaluated using Monte Carlo hypothesis testing

#### Spatio-temporal cluster in phase II

**Fig. 5:**
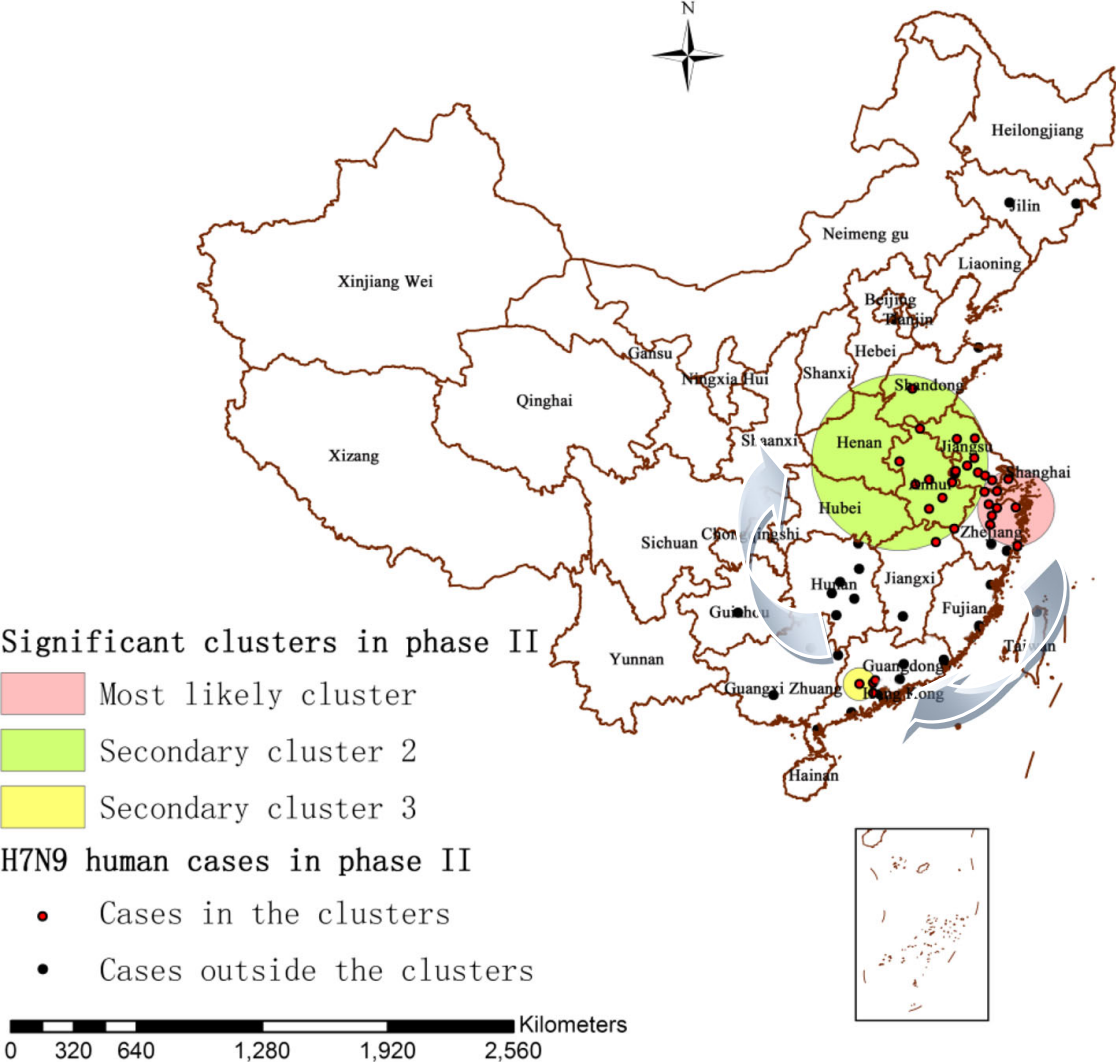
Significant spatio-temporal clusters (P < 0.05) of A(H7N9) human infections in China as identified by the space-time permutation scan statistic (phase II), which are illustrated by the most likely cluster (pink circle), a secondary cluster 2 (green circle) and a secondary cluster 3 (yellow circle). The blue arrows describe the chronological progression of clusters, and outbreaks represented by red and black dots are also shown

There were totally 72 localities and 294 influenza A(H7N9) human cases contained in the second epidemic phase. The identified statistically significant spatio-temporal clusters of A(H7N9) human infections from June 1, 2013 to May 31, 2014 (364 days) were displayed in Fig. 5, in which the correlated influenza A(H7N9) human cases in this period were overlaid too. In the first 6 months of the second H7N9 epidemic phase (between June 1, 2013 and December 31, 2013), there was no statistically significant cluster detected in China, and then the space-time test identified three significant clusters (*P* < 0.05), one most likely cluster and two secondary clusters (Fig. 5 and Table 3). The most likely cluster (*P* = 0.0016) including 10 localities was identified in Zhejiang province, Jiangsu province and Shanghai municipality between January 12, 2014 and January 31, 2014 with a radius of 194.90 km, which showed 61 influenza A(H7N9) human cases accounting for 20.75% of all the human cases of phase II (61/294), when only 33.80 were expected theoretically. The secondary cluster 2 (*P* = 0.011) including 18 localities was identified in Anhui, Jiangsu, Shandong and Jiangxi provinces between April 22, 2014 and May 31, 2014, with a radius of 453.14 km, including 4.76% of the total human cases of this phase (14/294) whereas only 3.58 could be expected. The secondary cluster 3 (*P* = 0.028) including 4 localities was identified in Guangdong province between February 16, 2014 and March 2, 2014, with a radius of 80.49 km. This cluster included 6.46% of the total human cases of this phase (19/294) when only 6.53 could be expected theoretically.

**Table 3:** Statistically significant spatio-temporal clusters of A(H7N9) human infections in China identified by use of the space-time permutation scan test with 5-days scanning unit (phase II)

Cluster	Most likely cluster	Secondary cluster 2	Secondary cluster 3
Location	Ningbo, Shaoxing, Jiaxing, Zhuji, Hangzhou, Shanghai, Yiwu, Huzhou, Suzhou, Taizhou	Fuyang, Lu’an, Hefei, Xuzhou, Anqing, Tongling, Ma’anshan, Xuancheng, Nanjing, Huaian, Zhenjiang, Taian, Taizhou, Yancheng, Changzhou, Wuxi, Huangshan, Leping	Zhaoqing, Foshan, Jiangmen, Guangzhou
Province or region	Zhejiang, Jiangsu, Shanghai	Anhui, Jiangsu, Shandong, Jiangxi	Guangdong
Coordinates	29.86 N, 121.56 E	32.89 N, 115.81 E	23.06 N, 112.45 E
Radius (km)	194.90 km	453.14 km	80.49 km
Time frame	2014 1/12–1/31	2014 4/22–5/31	2014 2/16–3/2
Duration (days)	19	39	14
O^a^	61	14	19
E^b^	33.80	3.58	6.53
O / E^c^	1.80	3.91	2.91
LLR^d^ / Test statistic	10.292886	8.856121	8.095875
P-value^e^	0.0016	0.011	0.028

^a^Number of cases observed per cluster

^b^Number of cases expected per cluster

^c^Observed / Expected, Observed-to-expected ratio

^d^Log Likelihood ratio

^e^Statistical significance was evaluated using Monte Carlo hypothesis testing

#### Spatio-temporal cluster in phase III

Although there were still 36 influenza A(H7N9) human cases occurred in 21 localities during the third epidemic phase (213 days), there was no statistically significant spatio-temporal cluster of A(H7N9) human infections (*P* < 0.05) identified in phase III (June 1, 2014 to December 31, 2014) using the space-time permutation model.

By using the space-time permutation model, a total of five statistically significant spatio-temporal clusters (P < 0.05) were identified during the study period, in which two clusters were observed in phase I, another three clusters were observed in phase II, respectively, and there was no significant cluster occurred in phase III. In the three phases, around 29.35% of the total influenza A(H7N9) human cases were located in the five significant spatio-temporal clusters identified in this study (135/460), the radius of these clusters varied between 80.49 km and 596.94 km.

## Discussion

The emergence of A(H7N9) human infections in China has posed a great threat to both the society and the public. How H7N9 outbreaks spread is not clear till now. Although some H7N9 family clusters have been reported in China [30–32], so far there is no evidence of sustained or efficient human-to-human H7N9 virus transmission [33–35]. As a complementary instrument of public health surveillance, geographical analysis can investigate spatial distribution of a disease within a territory [36]. In order to thoroughly examine the spatial-temporal spreading process of A(H7N9) human infections between March 2013 and December 2014, we investigated the spatial and temporal distribution, the directional trend and the statistically significant spatio-temporal cluster of A(H7N9) human infections by using a range of GIS and space-time statistical methods in this study.

A former research showed that some areas of Eastern China have a higher risk of infection with non-uniform distribution of H7N9 outbreaks [37]. However, our results suggested that the high incidence areas of H7N9 epidemic were mainly located in the eastern and the southeast coast of China, such as Shanghai, Zhejiang, Guangdong and Jiangsu. This difference may be due to a longer study period in our paper and the epidemic has changed as time went on. It is noteworthy that the similar living and geomorphological environments in Shanghai, Zhejiang, Guangdong and Jiangsu may be suitable for the virus spreading. Since a host of the affected cities were the most densely developed middle- and large-sized cities, the population density may associate with the spread of H7N9, consisting with a previous research [38]. In addition, we also noted that there were 9 new influenza A(H7N9) human cases occurred in Xinjiang between July 2014 and December 2014, where away from the eastern and the southeast coast of China and no case had reported before, this phenomenon is of concern and merits further investigation.

Influenza A(H7N9) human infections may have strong epidemic characteristics due to the seasonal pattern showed in Fig. 2. The epidemic was mainly concentrated between January and April, which suggested there was a possible seasonally higher risk of A(H7N9) human infections in China. This also provided some useful insights into H7N9 outbreaks in humans that the high incidence stage might coincide with the dry season months of January to April in China, and the occurrence of the epidemic peak was likely related to increased virus survival at rising temperature in this period. Some previous researches have highlighted that relative humidity and temperature may contribute to the occurrence of A(H7N9) human infections [15, 38], and an optimal temperature between 7 °C and 15 °C may be one of the driving forces for H7N9 [39]. Consequently, three epidemic phases were identified in this study (Fig. 2): (1) phase I was the initial outbreak stage of A(H7N9) human infections which lasted 79 days, and the sudden outbreak of H7N9 epidemic then resulted in a large number of influenza A(H7N9) human cases (*n* = 130). However, it is notable that few human cases were reported in the summer of 2013 after some suitable public health measures such as closure of live poultry markets which announced and implemented by the government [40, 41], and this confirmed that suspension of live poultry trading was an effective measure to contain H7N9 outbreaks in humans [42]. Furthermore, the existing researches also showed a possibility that live poultry markets contaminated by H7N9 virus were the most likely sources of the epidemic [6, 38, 43, 44]. And (2) in the beginning of phase II, there was an obvious reduction in the number of influenza A(H7N9) human cases and the low occurrence of the desease lasted nearly 6 months. But H7N9 outbreaks in humans resurged in the winter of 2013 [43], from January 2014 the number of the human cases began to sharply increase. And last (3) in phase III, only a slight rising but no obvious decline tendency of influenza A(H7N9) human cases’ number was observed due to the limitation of the research data in this study. This might because that the third phase was in an incubation period of the next epidemic curve, and the recurring H7N9 outbreaks in humans called for more improved preventive and control measures in China in the future.

In the first epidemic phase, the ellipse of influenza A(H7N9) human cases was the smallest one during the study period, which suggested that there were only some local H7N9 outbreaks in humans appeared in limited affected areas in these early days of the epidemic, especially some areas in eastern China. Moreover, the first epidemic phase displayed a more regular circular distribution than the others with a slight northwest-southeast elongation, and there was no obvious difference between the long axis and the short axis, which revealed that the directional trend of the epidemic was not apparent at the beginning. Although the spread of the outbreaks was limited then, there still appeared two significant spatio-temporal clusters of the epidemic. The secondary cluster 2 (*P* = 0.044) first appeared in phase I, with more influenza A(H7N9) human cases (*n* = 31), closer geographical distance between the human cases (radius = 271.46 km) and longer duration time (29 days) than the most likely cluster of phase I. An army of influenza A(H7N9) human cases gathered within small geographic scope in this cluster between March 13, 2013 and April 11, 2013, this was probably a consequence of the sudden outbreak of H7N9 and the lack of prevention measures in these early days of the epidemic. Subsequently, the most likely cluster (*P* = 0.0000055) appeared, which was the largest identified cluster (radius = 596.94 km) during the study period. Furthermore, over time the cluster shifted from the east to the southeast, which inferred that the epidemic might have a possible trend of spreading to the southeast coast of China.

In the second epidemic phase, both directional trend and local spatio-temporal clusters of H7N9 outbreaks in humans changed greatly. From the beginning of phase II (June 2013), influenza A(H7N9) human cases appeared sporadically for almost 6 months. But then a large number of influenza A(H7N9) human cases started appearing in the east and southeast of China between January 2014 and May 2014, which forming a larger ellipse and three significant spatio-temporal clusters of A(H7N9) human infections in this phase. The mean center (phase II) moved southwestward indicating that the H7N9 virus had spread along the southeast coast of China and hence the endemic area had shifted southwesterly. Furthermore, the standard deviational ellipse around the areas of A(H7N9) human infections substantially increased, suggesting that the infected areas had become larger than before. We also noted that the orientation of the ellipse had decreased from 152.621015 (phase I) to 26.947956 (phase II), indicating that the directional trend of A(H7N9) human infections had transformed from northwest-southeast to southwest-northeast direction. The long axis grew more significantly with an extending in the southwest-northeast directions and its length was nearly 2.7 times the short axis, which implied that the epidemic mainly spread along the southeast coast of China with a strong coastal growth. In addition, three statistically significant spatio-temporal clusters were observed in this phase, which shifted from the east to the southeast, and then back to the east-central China, and this suggested a possible epidemic trend of spreading to central China. The most likely cluster of phase II (*P* = 0.0016) contained the most influenza A(H7N9) human cases of all clusters detected in the three phases (*n* = 61). Then the secondary cluster 3 (*P* = 0.028) was observed in Zhaoqing, Foshan, Jiangmen and Guangzhou, and these four cities all belong to Guangdong province, suggesting that Guangdong was a high incidence area of A(H7N9) human infections in space - time dimension at that time. As the season progressed, the largest cluster of phase II developed in Anhui, Jiangsu, Shandong and Jiangxi provinces (Secondary cluster 2). Compared with the previous two clusters identified in phase II, this cluster showed the least influenza A(H7N9) human cases (*n* = 14), the largest geographical range (radius = 453.14 km) and the longest duration time (39 days) in this phase. This indicated that the number of the human cases decreased, but there was still an obvious trend of spatial diffusion of the epidemic.

However, there was no significant spatio-temporal cluster of A(H7N9) human infections identified during the third epidemic phase. This may be caused by a slice of sporadic influenza A(H7N9) human cases reported in the second half of 2014 and the relatively long period (213 days) with low H7N9 virus activity in this period. In phase III, the mean center of standard deviational ellipse moved from southeast to central regions, suggesting a further disease spreading throughout central China. Moreover, the size of the ellipse increased continuously, accordingly the geographical size of H7N9 outbreaks in humans reached the maximum value of the three phases. We observed that the orientation of the ellipse increased to 123.918912°, meanwhile, the long axis increased obviously with a northwest-southeast elongation and its length was nearly 3.66 times the short axis, all of this indicated that the directional trend of A(H7N9) human infections was more focused on the northwest-southeast with a strengthened spreading force, signaling a strong inland growth. Consequently, the significant reduction of the number of A(H7N9) human infections and the further expansion of the epidemic all contributed to the disappearance of the spatio-temporal cluster in phase III.

Although A(H7N9) human infections had not spread across the whole country, the epidemic moved from east to southeast coast, and thence to central China as the time changed, suggesting a future epidemiological trend that H7N9 outbreaks in humans would likely to continue and disperse to more central regions, and a few new human cases might appear in parts of the western China too. Meanwhile, the continuous increase in the size of the standard deviational ellipse signifying the relentless expansion of the infected area warned that the disease transmission cycle had not been interrupted yet. Surveillance and regional disease control preparedness should be strengthened accordingly. Furthermore, the disappearance of the cluster in phase III confirmed that the spatial-temporal clustering of A(H7N9) human infections in China between 2013 and 2014 presented different forms in different areas and times [3].

There are three potential limitations in this paper. Firstly, our analyses did not include the data of influenza A(H7N9) human cases after 2014 because which were either not available or imprecise. Secondly, this study has mainly examined the spatiotemporal spread patterns of A(H7N9) human infections in China between March 2013 and December 2014, further researches are still required to investigate the ecological risk and delineate the epidemiological nature of A(H7N9) human infections in the future. Thirdly, because the retrospective space-time permutation scan statistic model for detecting of disease outbreaks only uses case numbers and does not require the population-at-risk data, so the association of the spatio-temporal clusters of A(H7N9) human infections with population density has not been assessed in this study. However, the spatio-temporal clusters of the epidemic might still be associated with the population density of the study areas, which will be further studied in our future research. In addition, although we cannot get the complete and accurate data of influenza A(H7N9) human cases after 2014, the case reports in World Health Organization website [4] showed that H7N9 outbreaks in humans between January 2015 and August 2017 presented obvious seasonality and regionality: Winter–spring season was the most prevalent period for H7N9, and most cases were concentrated in the eastern and the southeast coast of China; with the spreading of the epidemic, human cases in central China have increased significantly, and a small number of new human cases have also been reported in parts of the western regions of China. These phenomena are consistent with the epidemiological trend predicted in this study, therefore our results are credible and reasonable, which can lay a foundation for not only risk-based surveillance but also investigations into risk factors associated with A(H7N9) human infections, and also can provide some meaningful information to future researches on assessing the transmission mechanism of H7N9 in China.

## Conclusions

A(H7N9) human infections has essentially become a severe public health threat in China for several years. In order to explore the spreading dynamics of the epidemic, a phased assessment of the potential directional trend and the significant spatio-temporal clustering of H7N9 outbreaks in humans were investigated in this study. Consequently, three standard deviational ellipses and five significant spatio-temporal clusters (*P* < 0.05) of the epidemic were identified during the study period. The results showed that A(H7N9) human infections started in eastern China, persisted for a long period in southeast coastal areas, and then dispersed throughout the central regions, with a trend of continuous spatial expansion. Furthermore, the epidemic was space-time clustered during the first two phases in China, but it was randomly distributed in phase III. In order to limit the spread of the outbreaks, surveillance activities, closure of live poultry markets, and health education should be selectively taken within highly endemics areas in the epidemic season (especially between January and April). In short, this study can provide valuable references for establishing effective monitoring and preventive measures and controlling the virus spread during future H7N9 epidemic in China.

## Abbreviations

CenterX,: X coordinate for the mean center
CenterY,: Y coordinate for the mean center
XStdDist: The standard distance of X-axis
YStdDist: The standard distance of Y-axis
